# Association between common laboratory indices and IgAV recurrence in children

**DOI:** 10.1186/s12887-022-03657-9

**Published:** 2022-10-19

**Authors:** Juan Zhou, Li Li, Jing Luo, Yingtian Yang, Xing Shen

**Affiliations:** 1 Department of Pediatrics, The Affiliated Hospital of Southwest Medical University, 646000 Luzhou, Sichuan China; 2 Department of Pediatrics, Bazhong Central Hospital, 636000 Bazhong, Sichuan China; 3 Department of Endocrinology, Bazhong Central Hospital, 636000 Bazhong, Sichuan China

**Keywords:** Common laboratory indices, Children, IgAV, Recurrence

## Abstract

**Background:**

IgA vasculitis (IgAV) is a common type of vasculitis seen in children. IgAV recurrence can result in chronic kidney disease. We aimed to explore the association between common laboratory indices and IgAV recurrence in children, and to establish a prediction model.

**Methods:**

This retrospective study included children with diagnosed with IgAV hospitalized in Bazhong Central Hospital, Sichuan, from January 2014 to December 2019. Children were assigned to two groups based on IgAV recurrence, and baseline clinical data were collected for comparison. A logistic regression model to predict IgAV recurrence was established. The receiver operating characteristic curve was plotted. The area under the curve (AUC) was used to detect performance of the predictive model.

**Results:**

This study included 193 children (39 [20.2%], recurrence group; 154 [79.8%], non-recurrence group). Based on multivariate regression analysis, the duration of illness and joint involvement were independent predictors of IgAV recurrence in children (*P* < 0.05). No significant differences were observed in common laboratory indices (*P* > 0.05). The AUC of the prediction model was 0.766 (*P* < 0.001) with sensitivity of 74.4% and specificity of 68.8%.

**Conclusion:**

Common laboratory indices were not associated with recurrence of IgAV in children.

## Background

IgA vasculitis (IgAV) is a common type of vascular inflammation that mainly affects children; however, its cause and pathogenesis remain poorly understood. It also affects adults, but the incidence of the disease is high in children (6–22 per 100,000 person-years). Although it usually has a favorable prognosis, the recurrence rate is high in children (2.7–66.2%)[1]. The prevalence of IgAV is higher in males than females. It is most commonly seen in children aged 5–7 years. Moreover, the disease onset is more frequent in the winter season [2, 3]. The main clinical manifestations of IgAV include nonthrombocytopenic purpura, arthritis or arthralgia, abdominal pain, gastrointestinal bleeding, and nephritis [4], and the severity of kidney injury is the key determinant for the prognosis of IgAV in children [4, 5]. Some studies have revealed that recurrence of IgAV in children can lead to kidney involvement or exacerbation of kidney injury [6, 7]. Therefore, early prediction and interventions in recurrence of IgAV have important clinical implications.

Presently, there is no consensus regarding the findings on prediction of IgAV recurrence, and no specific routine laboratory test is available for the diagnosis of IgAV. Common laboratory indices, such as C-reactive protein (CRP), procalcitonin, white blood cell (WBC) count, and red blood cell (RBC) count are evaluated to rule out other diseases[8]. Results of coagulation studies are usually in the normal range in IgAV patients. Few studies revealed platelet count and mean platelet volume (MPV) as the only laboratory indices related to IgAV recurrence. Low levels of platelet count and high MPV values were observed. Inflammatory markers, such as CRP and erythrocyte sedimentation rate (ESR) appear to be elevated [9–15]. Inflammatory mechanisms play a major role in the etiology of vascular diseases.

Neutrophil/lymphocyte ratio (NLR) is a serum marker for inflammatory response and therefore, it is used in systemic inflammatory diseases. Moreover, NLR is inexpensive and easy to assess. Therefore, NLR is useful in evaluating the severity of the disease [16]. Existing studies have shown that NLR and CRP have higher predictive value for IgAV recurrence in adults [17]; however, few data are available on similar findings in children. Therefore, in the present study, we aimed to explore whether the common laboratory indices could predict IgAV recurrence in children and provide an accessible theoretical basis for early intervention to prevent recurrence of IgAV in children.

## Methods

### Participants

The retrospective study included children who were initially diagnosed with IgAV and admitted in the Pediatrics Department at Bazhong Central Hospital, Sichuan, China from January 2014 to December 2019. The research related to human use has been complied with all the relevant national regulations, institutional policies and in accordance the tenets of the Helsinki Declaration, and has been approved by the Medical Ethics Committee of Bazhong Central Hospital. All family members of children enrolled in this study gave informed consent.

Children diagnosed with IgAV according to criteria jointly developed by the European League Against Rheumatism (EULAR), Pediatric Rheumatology International Trials Organization (PRINTO), Pediatric Rheumatology European Society (PRES) in 2010 [4] ,and European consensus-based recommendations for diagnosis and treatment of immunoglobulin A vasculitis [18] were included in the study. These criteria were the presence of nonthrombocytopenic palpable (necessary) skin purpura accompanied by any of the following conditions: [1] acute diffuse abdominal pain; [2] leukocytoclastic vasculitis with immunoglobulin A (IgA) immune complex deposition being predominant or glomerulonephritis with IgA deposition being predominant in the histological examination; [3] arthritis or arthralgia; and [4] manifestations of kidney injury: 24-hour urine protein quantification > 0.3 g or albumin–creatinine ratio > 30 mmol/mg in the morning urine sample and red blood cells per high-power field ≥ 5 or urine occult blood ≥ 2 + or urine sediments showing red cell cast. The exclusion criteria were [1] no initial diagnosis of IgAV before the present study; [2] received drugs affecting platelet, coagulation function, hematopoietic function, and immune function within the past 2 weeks; [3] presence of blood system diseases; [4] presence of severe diseases of heart, liver, kidney, and other organs; [5] inability of children or their family members to cooperate with treatment; and [6] incomplete data. After the initial diagnosis of IgAV in children, if relevant clinical symptoms reoccurred after at least 4 weeks since the disappearance of primary symptoms, recurrence was confirmed, and such children were allocated to the recurrence group. However, children who did not present with a relapse after the stipulated time were allocated to the non-recurrence group.

## Data collection

In this study, we collected various demographic and clinical data of patients, including gender; age; duration of illness; use of CS; organ involvement; duration of treatment; presence of respiratory tract infection; and various clinical laboratory indices, such as WBC count, neutrophil (NEU) count, lymphocyte (LYM) count, platelet (PLT) count, MPV, RBC count, hemoglobin (HGB) level, monocyte (MONO) count, eosinophils (EOS) count, basophils (BAS) count, and CRP. Moreover, we calculated NLR and platelet/lymphocyte ratio (PLR).

### Statistical analysis

Statistical analyses were performed using SPSS, version 25 (IBM Corp., Armonk, NY, USA). Normally distributed measurement data were presented as mean ± standard deviation and evaluated using the Student’s t test. Non-normally distributed data were presented as median and quartile *M (P25, P75*) and evaluated using the Mann–Whitney U test. Count data were presented as number (n) and percentage (%) and evaluated using the χ^2^ test. Multivariate logistic regression analysis (backward) was performed to establish the prediction model of IgAV recurrence. A receiver operating characteristic (ROC) curve was plotted to evaluate the performance of the prediction model by determining the optimal threshold with the highest sensitivity and specificity for predicting IgAV recurrence. A *P*-value <0.05 was considered statistically significant.

## Results

### Characteristics of patients

In total, 193 children diagnosed with IgAV were enrolled in the study. Of these, 39 (20.2%) children were assigned to the recurrence group and 154 (79.8%) children to the non-recurrence group, indicating a recurrence rate of 20.2%. The presence of relevant clinical symptoms again at least 4 weeks after remission of IgAV was considered recurrence of IgAV.

The baseline characteristics of children enrolled in the recurrence and non-recurrence groups are given in Table 1. The mean ages of children in the recurrence and non-recurrence groups were 6 and 7 years, respectively. The duration of illness in the recurrence group was longer than that in the non-recurrence group (5.0 (3.0, 10.0) vs. 3.0 (2.0, 5.3), *P <* 0.05). However, there were no statistically significant differences in terms of age; gender; organ involvement; duration of treatment; presence of respiratory tract infection; use of CS; and laboratory indices, such as WBC count, RBC count, NEU count, LYM count, MONO count, EOS count, BASO count, MPV, NLR, PLR, CRP, and PLT between the groups (*P >* 0.05).

**Table 1:** Comparison between clinical characteristics of children with IgAV in recurrence and non-recurrence groups

Items		Recurrence group (n = 39)	Non-recurrence group (n = 154)	*P*
Age (years)		6.0 (5.0, 9.0)	7.0 (5.5, 9.0)	0.357
Gender, n (%)	Male	19 (48.7)	73(47.7)	0.883
	Female	20 (51.3)	81 (52.6)	
Other involved systems except skin, n (%)	Joint	27(69.2)	89(57.8)	0.193
	Gastrointestinal tract	21 (53.8)	21 (53.8)	0.823
	Kidney	3 (7.7)	7 (4.5)	0.428
Duration of illness (d)		5 (3.0, 10.0)	3 (2.0, 5.3)	0.000
Duration of treatment (d)		6 (4.0, 8.0)	5(4.0, 7.0)	0.155
Presence of respiratory tract infection, n (%)		8 (20.5)	51 (33.1)	0.127
Use of CS, n (%)		31 (79.5)	112 (72.7)	0.389
Laboratory indices:				
WBC (×10^9^/L)		9.78 (9.06, 11.04)	10.29 (7.53, 13.63)	0.508
NEU (×10^9^/L)		6.90 (5.57, 8.08)	6.71 (4.43, 10.20)	0.802
LYM (×10^9^/L)		2.30 (1.89, 2.86)	2.41 (1.76, 3.25)	0.594
PLT (×10^9^/L)		317.00 (265.00, 370.5)	311.00 (249.00, 376.00)	0.669
MPV (fl.)		10.60 (9.79, 11.10)	10.20 (9.20, 11.60)	0.417
RBC (×10^12^/L)		4.39 (4.11, 4.54)	4.50 (4.25, 4.72)	0.060
HGB (g/L)		121.00 (115.50, 128.00)	125.00 (118.00, 131.00)	0.078
CRP (mg/L)		4.60 (3.15, 14.50)	4.20 (2.00, 12.00)	0.244
NLR		2.89 (1.89, 4.35)	2.69 (1.64, 4.86)	0.728
PLR		126.52 (98.74, 172.42)	129.36 (87.05, 175.78)	0.410
MONO(×10^9^/L)		0.63 (0.42,0.77)	0.58 (0.38,0.74)	0.517
EOS(×10^9^/L)		0.08 (0.01,0.20)	0.07 (0.01,0.18)	0.872
BASO(×10^9^/L)		0.01 (0.01,0.02)	0.02 (0.01,0.03)	0.130

Data were presented as number (percentage), mean ± standard deviation, or median (interquartile range)

CS, corticosteroid; CRP, C-reactive protein; HGB, hemoglobin; LYM, lymphocyte; MPV, mean platelet volume; NEU, neutrophil; NLR, neutrophil/lymphocyte ratio; PLR, platelet/lymphocyte ratio; PLT, platelet; RBC, red blood cell; and WBC, white blood cell; MONO: monocyte; EOS, eosinophils; BASO, basophils

*P* < 0.05 is considered statistically significant

### Prediction model of IgAV recurrence

**Fig. 1:**
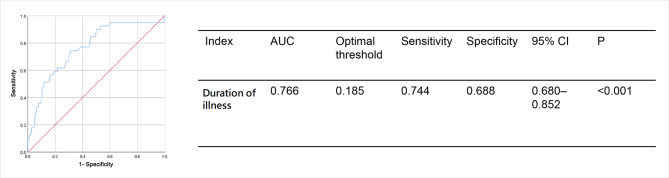
ROC curve of the duration of illness for prediction of IgAV recurrence The table shows the AUC values for the duration of illness. AUC, Area under the curve; CI, confidence interval

The risk factors for IgAV recurrence were evaluated using univariate and multivariate logistic regression analyses. Univariate analysis showed that the duration of illness (odds ratio [OR]: 1.169, 95% confidence interval [CI]: 1.074—1.272) was associated with IgAV recurrence in children. Furthermore, multivariate analysis indicated that the duration of illness (OR: 1.198, 95% CI: 1.092–1.315, *P* < 0.001) and joint involvement (OR: 2.596, 95% CI: 1.097–6.147, *P* = 0.030) were independent predictors for IgAV recurrence (Table 2).No significant differences were observed in duration of treatment,use of CS, presence of respiratory tract infection, and other laboratory indices, such as WBC, NEU, LYM, PLT, MPV, RBC, HGB, NLR, and PLR (*P* > 0.05). According to the ROC curve analysis of the duration of illness, an optimal threshold of 0.185 with sensitivity of 74.4% and specificity of 68.8% was observed. The area under the curve (AUC) of the prediction model was 0.766 (an acceptable discrimination; 95% CI: 0.680–0.852; *P* < 0.001; Fig. 1).

**Table 2:** Univariate and multivariate regression analysis for evaluating risk factors for IgAV recurrence in children

Variables	Univariate analysis		Multivariate analysis	
	OR (95% CI)	*P*	OR (95% CI)	*P*
Duration of illness	1.169 (1.074, 1.272)	0.000	1.198 (1.092, 1.315)	0.000
Duration of treatment	1.105 (0.978, 1.249)	0.110	1.142 (0.999, 1.306)	0.052
Presence of respiratory tract infection	0.521 (0.224, 1.215)	0.521	0.308 (0.113, 0.839)	0.210
Joint involvement	1.643 (0.775, 3.484)	0.195	2.596 (1.097, 6.147)	0.030
Use of CS	1.466(0.624,3.446)	0.391		
WBC	0.949 (0.863, 1.042)	0.949		
NEU	0.957 (0.867, 1.057)	0.385		
LYM	0.868 (0.632, 1.192)	0.382		
PLT	1.000 (0.997, 1.004)	0.814		
MPV	1.051 (0.853, 1.296)	0.639		
RBC	0.413 (0.162, 1.053)	0.064		
HGB	0.971 (0.943, 1.000)	0.053		
NLR	0.987 (0.927, 1.050)	0.675		
PLR	1.001 (0.996, 1.006)	0.591		
MONO	1.194(0.405,3.522)	0.748		
EOS	0.684(0.126,3.715)	0.660		
BASO	0.190(0.000,45866.481)	0.793		

CS, corticosteroid; HGB, Hemoglobin; LYM, lymphocyte; MPV, mean platelet volume; NEU, neutrophil; NLR, neutrophil/lymphocyte ratio; OR, odds ratio; PLR, platelet/lymphocyte ratio; PLT, platelet; RBC, red blood cell; and WBC, white blood cell;MONO: monocyte; EOS, eosinophils; BASO, basophils

*P* < 0.05 is considered statistically significant

## Discussion

IgAV is an IgA-mediated systemic vasculitis occurring mainly in children. The IgA deposition in the small blood vessels results in the symptoms of IgAV, affecting the kidneys, skin, and joints. Usually, IgAV has an excellent outcome in children. However, renal involvement (IgA vasculitic nephritis) at disease onset is the most severe complication of IgAV, affecting its prognosis. This renal involvement can result in end-stage kidney disease in children. Kidney injury has a higher incidence rate in children with recurrent IgAV than that in children with the initial episode of IgAV [2, 19–21]. Therefore, reducing IgAV recurrence is the need of the hour. In this retrospective study, we explored the association between common laboratory indices and IgAV recurrence in children for evaluating the predictors for IgAV recurrence. Findings of this study revealed that the duration of illness (OR: 1.198, 95% CI: 1.092–1.315) and joint involvement (OR: 2.596, 95% CI: 1.097–6.147) were independent predictors for IgAV recurrence in children. However, no significant differences were observed in any of the studied laboratory indices (*P* > 0.05). AUC of the prediction model was 0.766 (*P* < 0.05) with sensitivity of 74.4% and specificity of 68.8%.

Numerous foreign studies have confirmed that IgAV recurrence can result in kidney injury in children with IgAV and the recurrence rate ranges from 10.25 to 25% [11, 12, 22]. In the present study, we found that the recurrence rate was 20.2%, which was consistent with previous findings. Under no difference in treatment, the present study suggested that the duration of illness on initial diagnosis in the recurrence group was longer than that in the non-recurrence group, which was an independent risk factor for IgAV recurrence (*P* = 0.000), and consistent with findings of many other studies [23–26]. The reason for this finding may be that in autoimmune diseases, early treatment can promote the transformation from immune response involved by non-specific T cells and antibodies to that involved by specific T cells and antibodies as soon as possible, avoiding further tissue injury resulting from excessive activation of immune cells and release of more inflammatory factors [27].

The pathogenesis of IgAV in children remains unclear. Some studies have demonstrated that older age is a high risk factor for recurrence in the ethnic Chinese population; however, there are differences in the non-ethnic Chinese population [28]. In the present study, we did not observe the correlation between age and IgAV recurrence, which may be due to the limited small sample size or differences in allocation. Additionally, some studies have shown a correlation between different locations/systems, such as skin, kidney, joint, and gastrointestinal tract, or different numbers of these systems and IgAV recurrence [12]. In the present study, joint involvement was an independent predictive factor for IgAV recurrence (OR: 2.596, 95% CI: 1.097–6.147; *P* < 0.05). No other statistically significant differences were observed in location/systems involved and IgAV recurrence (*P* > 0.05), which may be because the clinical manifestations are only the result of immune inflammation, and its presence or absence and severity depend on the severity of the immune inflammation [15, 29–31]. Several studies reveal that infection, especially the upper respiratory tract infection, is a risk factor for patients with new-onset or recurrent IgAV [22]; however, in the present study, no correlation was observed between the presence of respiratory tract infection and IgAV recurrence in children (*P >* 0.05). Moreover, the present study suggested that there was no correlation between duration of treatment and IgAV recurrence (*P >* 0.05), which may be because IgAV is a self-limiting disorder, and its treatment only relieved symptoms or delayed the disease course.

Blood parameters, including NEU, PLT, LYM, monocyte (MONO), NLR, PLR, and monocyte/lymphocyte ratio (MLR), have been shown to be indices for systemic inflammation and infection [32]. Studies have shown that PLT and MPV are the only laboratory indices associated with IgAV recurrence in children [11]; however, the present study found that these common laboratory indices, on initial diagnosis in the recurrence group, did not differ significantly from those in the non-recurrence group (*P >* 0.05). Maybe because HSP is an IgA-mediated immune inflammation and not an inflammation caused by infection. Therefore, further research on differences in IgA and other immunoglobulinsare required to study the recurrence of IgAV in children.

This study has some limitations. First, the sample size was small. Second, the severity and duration of organ involvement have not been analyzed in detail. Therefore, further research is warranted to explore the association of IgA and other immunoglobulinswith the recurrence of IgAV in children.

In conclusion, the present retrospective study explored the predictive factors for IgAV recurrence in children. The recurrence rate was consistent with that of previous literature. The common laboratory indices were not associated with the recurrence of IgAV in children. The duration of illness and joint involvement were proved independent risk factors for IgAV recurrence. The duration of illness in the recurrence group was observed to be longer than that in the non-recurrence group. Therefore, clinical follow-up should focus on children with a duration from the onset of symptoms to admission of more than 4 days on initial diagnosis, and prevent IgAV recurrence.

## Data Availability

The datasets used and/or analysed during the current study are available from the corresponding author on reasonable request.

## List of abbreviations

IgAV: IgA vasculitis
CRP: C-reactive protein
WBC: white blood cell
RBC: red blood cell
MPV: mean platelet volume
ESR: erythrocyte sedimentation rate
NLR: Neutrophil/lymphocyte ratio
EULAR: European League Against Rheumatism
PRINTO: Pediatric Rheumatology International Trials Organization
PRES: Pediatric Rheumatology European Society
IgA: immunoglobulin A
NEU: neutrophil
LYM: lymphocyte
PLT: platelet
HGB: hemoglobin
PLR: platelet/lymphocyte ratio
MONO: monocyte
EOS: eosinophils
BASO: basophils
MLR: monocyte/lymphocyte ratio
CS: corticosteroid
